# Detection of Cyanide in a Decomposed Exhumed Body: A Case Report

**DOI:** 10.7759/cureus.62108

**Published:** 2024-06-10

**Authors:** Prasannan Kuniyil, Sujith Sreenivas C, Hemanth Mohan P V

**Affiliations:** 1 Forensic Medicine, Muslim Educational Society (MES) Medical College, Perinthalmanna, IND; 2 Forensic Medicine, Government Medical College, Kozhikode, Kozhikode, IND

**Keywords:** hydrocyanic acid, decomposed body, forensic toxicology, exhumed body, cyanide poisoning

## Abstract

Cyanide is a lethal poison that induces immediate fatality. Infrequently employed as a homicidal poison, it is not an ideal choice for homicide as it causes a ‘dramatic’ death causing suspicion among others. Cyanide is a rapidly metabolized poison that also rapidly disintegrates after death, posing challenges for chemical analysis, particularly when dealing with decomposed bodies. Detection of cyanide from a decomposed body is infrequent. A suspected case of intentional poisoning resulting in death was interred without conducting a postmortem examination. The exhumation process revealed the presence of hydrogen cyanide in the postmortem fluids collected from the body cavities three years after interment.

## Introduction

Cyanide is a toxic substance that undergoes metabolism and rapidly disintegrates in the body. The enzyme rhodanese converts it into thiocyanate [1]. Therefore, it is challenging to identify through chemical analysis when examining a deceased, particularly if an antidote has been administered or if there is a significant time lapse between death and sample collection. There is a lack of documented studies specifying the maximum postmortem intervals for identifying cyanide through chemical analysis in cases of poisoning. Detection of cyanide in experimental conditions and the time intervals are reported [2]. Cyanide detection in a decomposed body up to one year after death has been reported [3]. In the present case, hydrogen cyanide has been identified in the postmortem decomposition fluid extracted from the cavities of the interred body approximately three years following burial.

## Case presentation

**Figure 1:**
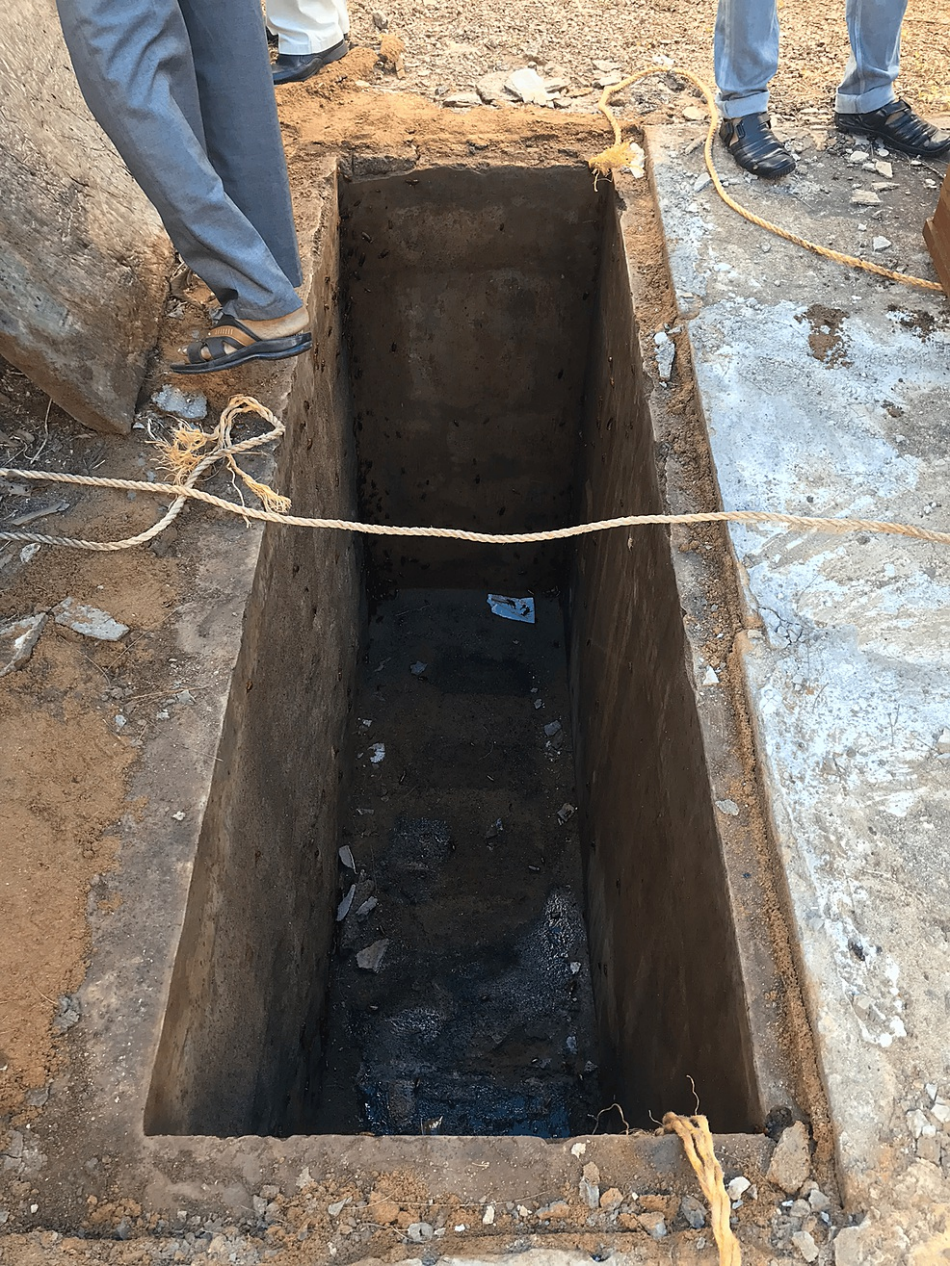
Image of the grave The grave was lined by laterite stones with cement plastering.

**Figure 2:**
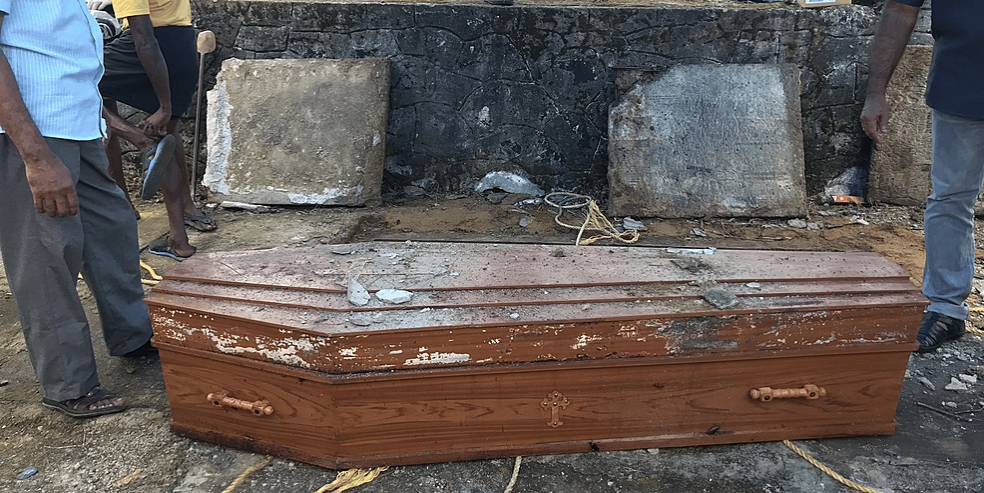
Image of the exhumed coffin box The body was in the wooden coffin, entombed beneath concrete slabs.

A 43-year-old woman collapsed outside a dentist's office in January 2016. She was taken to the nearest hospital and was found to be dead. Police intimation was not done as the ‘relatives’ alleged the cause as post epileptic, had no suspicion and the body was released to the relatives. The dead body was buried in the family grave in their church. Following a complaint from some other relatives later, an exhumation was performed in January 2019. The dead body was inside a wooden coffin with a lid, in a grave lined by laterite stones and a concrete slab covered it (Figures 1, 2).

**Figure 3:**
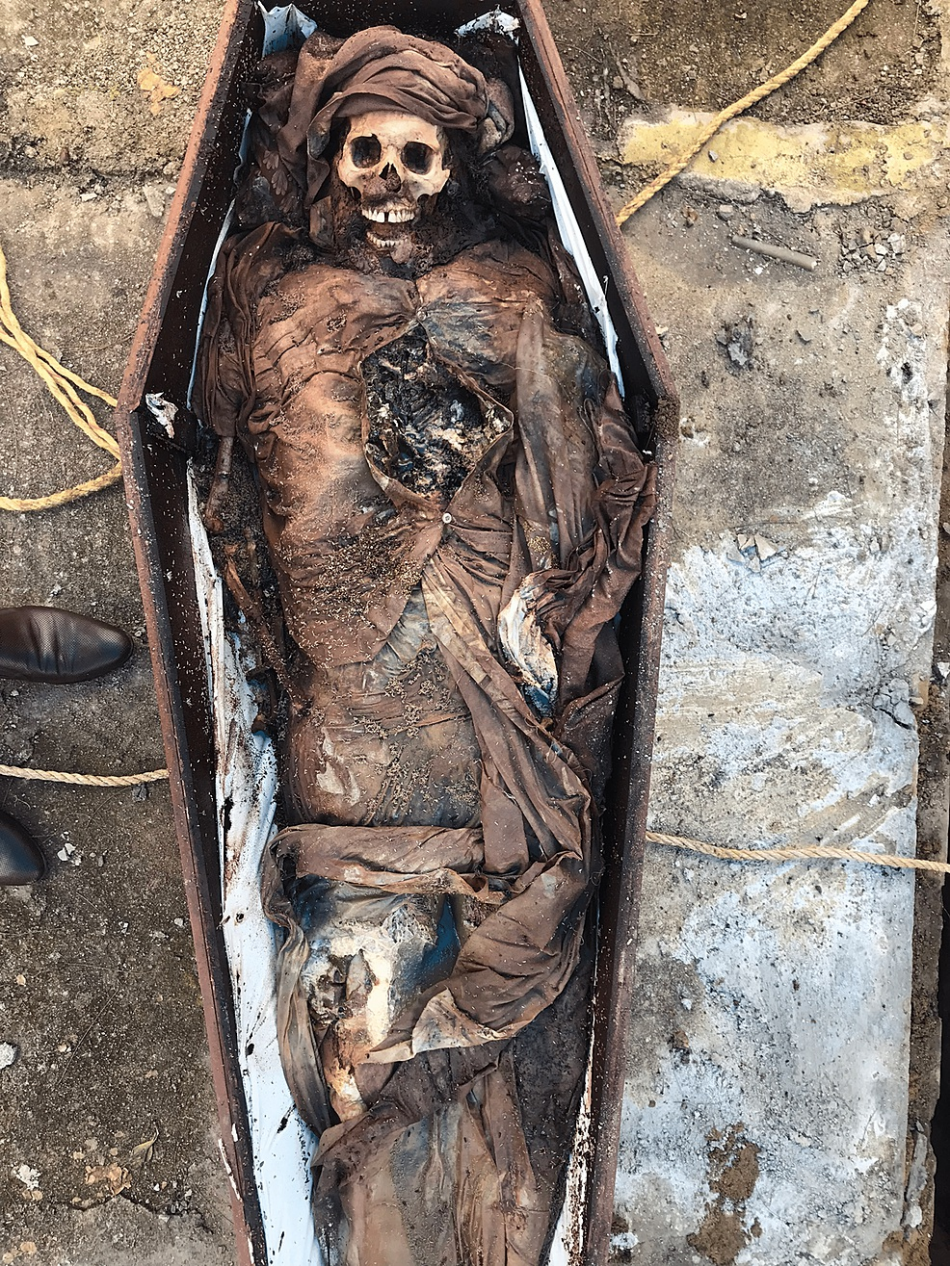
The skeletonized body inside the coffin

On examination, the body was skeletonized except for some soft decomposed scalp tissue at the back and side of the skull (Figure 3). Some amount of decomposition fluid was present inside the head and the trunk. The decomposition fluid, hair sample, one long bone (tibia) from the body, and soil sample below the coffin were collected for analysis.

The chemical analysis revealed the presence of hydrocyanic acid in the fluid collected from the trunk and the head. No poison was detected in the soil samples, the long bone (tibia), and the hair sample. Opinion was furnished as death due to cyanide poisoning. The accused was charged accordingly.

## Discussion

Cyanide is rarely used as a homicidal poison since it has a bitter taste and a characteristic smell (smell of bitter almonds or crushed Cassava leaves) [4]. The smell cannot be appreciated by about 20-40% of the population as the capacity to smell is a sex-linked recessive trait [1]. Chemical analysis of the viscera and body fluid is very vital for confirming the cause of death in cases of cyanide poisoning. Chemical analysis faces challenges in detecting cyanide due to its rapid disintegration in the body, particularly after decomposition. Detection of cyanide from a decomposed body is infrequent. Analysis conducted by the Regional Chemical Examiner's Laboratory within the Home Department, utilizing the alkaline picrate paper test method, has confirmed the presence of cyanide in the fluid remains discovered in both the head and trunk areas in this particular case. No traces of cyanide were found in the soil samples taken from the grave beneath the body. The preservation of cyanide may be attributed to the unique characteristics of the grave. It was lined with laterite stones and covered by a concrete slab. In this particular instance, this could potentially explain why the poison persisted for three years. Experimental in vitro studies showed that cyanide could be detected up to seven days after death [2]. Cyanide detection in a decomposed body one year after death has been reported [3].

The possibility of post-mortem cyanide formation in decomposing tissues should also be considered [5]. Though post-mortem generation of cyanide in decomposing tissues is rare, interference from the decomposition products would not produce erroneously high measurements [6].

## Conclusions

The presented case report underscores the notable discovery of cyanide in a significantly decomposed cadaver, even after a period of three years in a cemetery. The findings underscore the potential for identifying cyanide in a well-preserved highly decomposed body. While there is a chance of postmortem cyanide generation in decomposed tissues, its significance in forensic investigations is marginal. It is imperative to procure organs and tissues during the exhumation process, even in advanced decomposition cases. The retention of any decomposition fluid within body cavities is critical as it may facilitate the detection of rapidly metabolized toxins.
